# DscoreApp: A Shiny Web Application for the Computation of the Implicit Association Test D-Score

**DOI:** 10.3389/fpsyg.2019.02938

**Published:** 2020-01-10

**Authors:** Ottavia M. Epifania, Pasquale Anselmi, Egidio Robusto

**Affiliations:** Department of Philosophy, Sociology, Pedagogy, and Applied Psychology, University of Padova, Padua, Italy

**Keywords:** implicit association test, implicit measures, shiny, web application, *D-score*, user-friendly, social cognition

## Abstract

Several options are available for computing the most common score for the Implicit Association Test, the so-called *D-score*. However, all these options come with some drawbacks, related to either the need for a license, for being tailored on a specific administration procedure, or for requiring a degree of familiarity with programming. By using the R **shiny** package, a user-friendly, interactive, and open source web application (DscoreApp) has been created for the *D-score* computation. This app provides different options for computing the *D-score* algorithms and for applying different cleaning criteria. Beyond making the *D-score* computation easier, DscoreApp offers the chance to have an immediate glimpse on the results and to see how they change according to different settings configurations. The resulting *D-score*s are immediately available and can be seen in easy-readable and interactive graphs, along with meaningful descriptive statistics. Graphical representations, data sets containing the *D-score*s, and other information on participants' performance are downloadable. In this work, the use of DscoreApp is illustrated on an empirical data set.

## 1. Introduction

The Implicit Association Test (IAT; Greenwald et al., 1998) is one of the most common measures for assessing the strength of automatically activated associations between concepts. The resistance to self-presentation strategies (Egloff and Schmukle, 2002; Greenwald et al., 2009) and its ease of adaptation to different topics (Zogmaister and Castelli, 2006) make the IAT broadly used in studies on various issues, ranging from consumers behaviors (e.g., Karnal et al., 2016) and addiction behaviors (e.g., Chen et al., 2018) to self–esteem (e.g., Dentale et al., 2019) and personality traits (e.g., Steffens, 2004). Given its ability of overcoming self–presentation biases, the IAT finds many applications in social cognition studies, where it is employed for assessing implicit attitudes toward different social groups (e.g., Anselmi et al., 2015).

A convenient measure of the strength and direction of the implicit association assessed by the IAT is the *D-score* algorithm (Greenwald et al., 2003), for which different variations are available. The differences between each of the algorithms mainly concern the treatment for error and fast responses, while the core procedure for its computation is the same.

Despite many options are available for the *D-score* computation, like SPSS syntaxes, R packages, Inquisit scripts, they all come with some drawbacks. The use of SPSS syntaxes requires the SPSS license, programming skills are required for using R packages, and Inquisit scripts are tailored on Inquisit administration procedure. The aim of this study is to present an interactive Web Application for the computation of the *D-score* able to combine an easy and intuitive User Interface (UI) with the computational power of R, while being completely Open Source.

## 2. The Implicit Association Test—IAT

The IAT procedure (depicted in Table 1) is typically composed of seven different blocks, and is based on the speed and accuracy with which different type of stimuli (appearing sequentially at the center of the screen) are sorted in their reference categories (displayed at the top corners of the screen). Three blocks (Blocks 1, 2, and 5) are practice blocks, in which either object stimuli (e.g., images of *flowers* and *insects* in a Flowers-Insects IAT) or attribute stimuli (e.g., *Positive* and *Negative* words) are sorted in their reference categories. In the first associative condition (Blocks 3 and 4), flowers images and positive words are mapped with the same response key, while insects images and negative words are mapped with the opposite response key. In the second associative condition (Blocks 6 and 7), the labels for categorizing flowers and insects stimuli switch their positions on the screen. Thus, flowers images and negative words are mapped with the same response key, and insects images and positive words are mapped with the other response key. The categorization task is supposed to be easier in the condition consistent with respondents' automatically activated association (the so-called “compatible condition”) than in the condition against their automatically activated association (the so-called “incompatible condition”). In a more general fashion, the two associative conditions can be arbitrarily identified as Mapping A (e.g., Blocks 3 and 4) and Mapping B (e.g., Blocks 6 and 7). The difference between respondents' performance in the two conditions results in the *IAT effect* that can be easily interpreted by means of the *D-score*.

**Table 1:** IAT blocks and conditions, adapted from Greenwald et al. (2003).

**Block**	**Function**	**Left key**	**Right key**
B1	Practice	Flowers	Insects
B2	Practice	Good	Bad
B3	Practice Mapping A	Flowers + Good	Insects + Bad
B4	Test Mapping A	Flowers + Good	Insects + Bad
B5	Practice	Insects	Flowers
B6	Practice Mapping B	Insects + Good	Flowers + Bad
B7	Test Mapping B	Insects + Good	Flowers + Bad

*The presentation order of the critical blocks B3 and B4 and the critical blocks B6 and B7 is counterbalanced across participants*.

The IAT administration procedure might include a feedback strategy, for which a red cross appears on the screen every time a stimulus is incorrectly categorized. Participants are then asked to correct their response to continue the experiment.

### 2.1. The *D-Score*

The *D-score* algorithms result from the combination of the various error correction and lower tail treatment strategies (“Error inflation” and “Lower tail treatment” in Table 2).

**Table 2:** Overview of the *D-score* algorithms.

***D-score***	**Error inflation**	**Lower tail treatment**
D1	Built-in correction	No
D2	Built-in correction	Delete trials < 400 ms
D3	Mean (correct responses) + 2 sd	No
D4	Mean (correct responses) + 600 ms	No
D5	Mean (correct responses) + 2 sd	Delete trials < 400 ms
D6	Mean (correct responses) + 600 ms	Delete trials < 400 ms

*For all the algorithms, trials with a latency >10,000 ms are discarded. Trials from Blocks 3, 4, 6, and 7 are used for computing the D-score. Practice blocks (i.e., Blocks 1, 2, and 5) are discarded*.

Grounding on the IAT administration procedure, the error correction may apply either a built-in or an *ex post* correction. In the former case (*D1* and *D2*), the response time considered for the *D-score* computation is the time at the first incorrect response increased by the time required to correct it. In the latter case (*D3, D4, D5*, and *D6*), the error response is replaced by the average response time of the block in which the error occurred, increased by a fixed penalty (i.e., either 600 ms or two times the standard deviation of the block response time). The *D-score* algorithms differ also according to the lower tail treatment, which concerns the decision to discard fast trials (< 400 ms) or not. Once the treatments for the error and fast responses have been applied according to the chosen algorithm, the *D-score* can be computed. Firstly, the *D-score*s for associative practice blocks (Equation 1) and associative test blocks (Equation 2) are computed:

(1) $$\begin{array}{r} D_{\text{practice}}=\frac{M_{\text{B6}}-M_{\text{B3}}}{sd_{\text{B6, B3}}}, \end{array}$$

and

(2) $$\begin{array}{r} D_{\text{test}}=\frac{M_{\text{B7}}-M_{\text{B4}}}{sd_{\text{B7, B4}}}. \end{array}$$

In both cases, the difference in the average response times between the two critical blocks is divided by the standard deviation computed on the pooled trials of both blocks. Once the *D-score*s for practice and test blocks are obtained, it is possible to compute the actual *D-score*:

(3) $$\begin{array}{r} \text{D-score}=\frac{D_{\text{practice}}+D_{\text{test}}}{2}. \end{array}$$

The blocks order in Equations (1) and (2) is arbitrary, and can be reversed. The interpretation of the *D-score* clearly follows the order with which the subtraction between the blocks is computed. For instance, if the *D-score* of the Flowers-Insects IAT illustrated in Table 1 is computed following the blocks order in Equation 1 (*M*_B6_ − *M*_B3_) and Equation 2 (*M*_B7_ − *M*_B4_), a positive score would stand for a possible preference for flowers over insects (that is, faster responses would have been observed in B3 and B4 compared with B6 and B7). Vice versa, if the order of the blocks in Equation 1 and in Equation 2 is reversed (*M*_B3_ − *M*_B6_ and *M*_B4_ − *M*_B7_, respectively), a positive score would stand for a possible preference for insects over flowers.

Several options (illustrated in Table 3) are available for computing the *D-score*, namely Inquisit scripts, SPSS syntaxes, and R packages.

**Table 3:** Overview of the available options for computing the *D-score*.

	**Open source**	**Programming skills**	**Multiple D-score**	**Plot**	**Reliability**
SPSS syntaxes	No	A bit	Yes	No	No
Inquisit scripts	No	No	No	No	No
**IATanalytics**	Yes	Yes	Not clear	No	No
**IATScore**	Yes	Yes	Not clear	No	No
**IAT**	Yes	Yes	Yes	Yes	No
**IATScores**	Yes	Yes	Yes	Yes	Yes

*R packages are reported in bold*.

Inquisit scripts are probably the most straightforward way for obtaining the *D-score* since they compute it right after the IAT administration procedure and store the result along with other information on participants' performance (e.g., response time for each IAT trial, correct and incorrect responses). Nonetheless, these scripts work only when associated with the Inquisit administration procedure, and they can compute just one of the available *D-score* algorithms. Finally, Inquisit requires a license to be used.

SPSS syntaxes provides several information on participants' performance, and they are not tied to a specific administration software. Nonetheless, their use requires a certain degree of expertise with SPSS language, and SPSS license.

R provides the open-source alternative to both Inquisit scripts and SPSS syntaxes. Both **IATanalytics** and **IATScore** packages by Storage (2018a) and Storage (2018b) provide the users with just the function for computing the *D-score*. **IATScore** gives the chance to compute the score also for Brief-IAT (B-IAT; Sriram and Greenwald, 2009). Both **IAT** and IATScores provide functions for cleaning the original data set, for plotting the data, and for computing the different *D-score* algorithms. So far, only **IATScores** has built-in functions for computing IAT reliability (i.e., split–half and the test–retest IAT reliability).

Regardless of the specific R package one wants to use, the data preparation is not straightforward and easy. For some of the packages (e.g., **IATanalytics**), the columns identifying the variables for the computation of the *D-score* have to follow a specific order, otherwise the computation will fail. Also the coding of the variables might result counterintuitive: For example, in **IAT** package, error responses have to be coded as 1 and correct responses have to be coded as 0. Moreover, in both **IATanalytics** and **IATScore** it is possible to compute the *D-score* for just one participant at a time, and it is not well specified which *D-score* is computed. None of the above mentioned options provides the users with graphical representations of the *D-score*s.

An interactive tool able to combine a user–friendly interface with the computational power of R and its open source philosophy could represent an optimal solution for the *D-score* computation, also for researchers with no experience with coding. Additionally, this tool might be of convenience for researchers more experienced in coding and data analysis that want to obtain a quick overview of IAT results.

In the next sections, the functioning of DscoreApp is illustrated through a practical example.

## 3. The Chocolate-IAT Data Set

**Figure 1:**
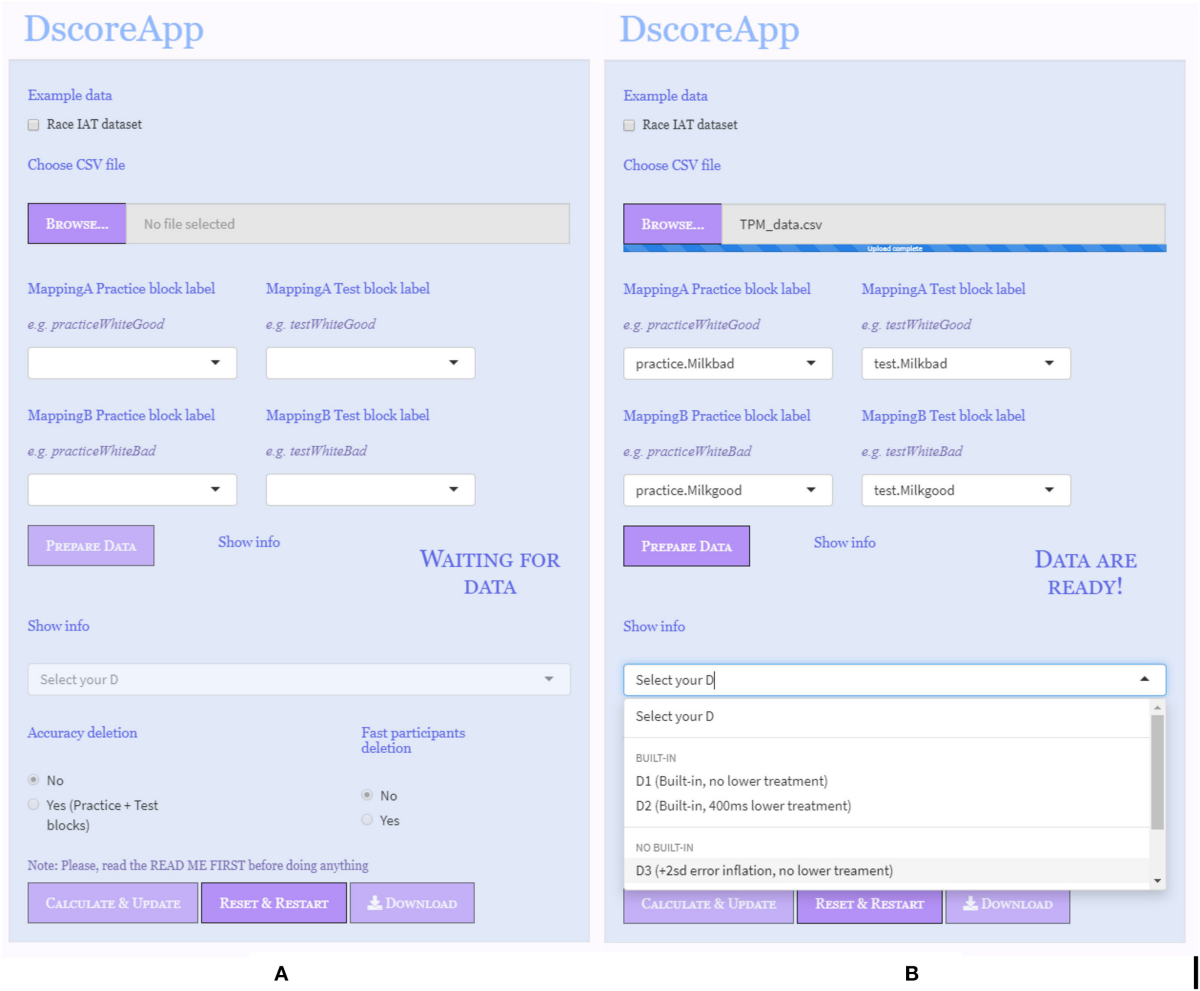
Input panel. **(A)** Data correctly uploaded. **(B)** Data ready for computation.

Data comes from the responses of 152 participants (*F* = 63.82%, Age = 24.03 ± 2.82) to a Dark-Milk Chocolate IAT. This IAT was developed for the assessment of dark and milk chocolate implicit preference. It followed the structure depicted in Table 1. The two critical conditions were made out of 60 trials each (i.e., 20 trials in each associative practice block and 40 trials in each associative test block). The associative condition in which Milk chocolate was associated with negative words and Dark chocolate was associated with positive words was identified as Mapping A (i.e., .Milkbad in Figure 1). Vice versa, the associative condition in which Dark chocolate was associated with negative words and Milk chocolate was associated with positive words was identified as Mapping B (i.e., .Milkgood in Figure 1). In case of an erroneous stimulus categorization, participants received no feedback. Results obtained from this data have been previously published in Epifania et al. (in press).

## 4. DscoreApp

DscoreApp was developed in R (R Core Team, 2018) by using **shiny** (Chang et al., 2018) and **shinyjs** (Attali, 2018) packages. DscoreApp can be retrieved at the URL: https://fisppa.psy.unipd.it/DscoreApp/, and its source code is available on GitHub. DscoreApp is platform independent, and is distributed under a MIT license. The UI is designed to be as clear and straightforward as possible, and the pop–up menus for the different functions are meant to make the use of the app more intuitive and interactive. The app is organized in different panels (i.e., “Input,” “Read me first,” “D-score results,” and “Descriptive statistics”) that will be presented in the next sections.

### 4.1. Read Me First Panel

The “Read Me First” panel includes important information regarding the app functioning. The interactive structure allows the users to jump directly to the instructions section they are interested in, making the information on the different app functions easily accessible. The **Download Template** button can be used to download a CSV template suggested for using the app. However, it is not strictly necessary to use the provided CSV template, or to specify the variables in the same order as in the template. As long as the uploaded file is in a CSV format (with comma set as separator) and contains the variables for the *D-score* computation with the same names as the variables in the CSV template, the app will work. Specifically, the data frame must contain a variable identifying participants' IDs (participant), the labels identifying the four critical blocks of the IAT (block), the latency of the responses expressed in milliseconds (latency), and the variable identifying the accuracy responses (correct).

The pure practice blocks (Blocks 1, 2, and 5) must be removed before using the app. If they are not removed, the app will throw an error. The block variable must be a character string that uniquely identify the four critical blocks of the IAT. This variable contains the information for distinguishing between the practice and test blocks of the two mapping conditions, such as “practiceMappingA,” “praticeMappingB,” “testMappingA,” and “testMappingB.” The specific name of each level is not important, as it is not important the order with which they have been presented to participants. In case the blocks labels are not unique, the app will throw an error. If the IAT administration procedure included a built-in correction, the latency variable must contain the already inflated response times. Otherwise, it must contain the raw response times. Finally, the correct variable must be a numeric variable with just two possible values, namely 0 identifying incorrect responses and 1 identifying correct responses. Usually, accuracy responses are automatically coded as 0 for incorrect and 1 for correct responses by the software for the IAT administration, unless otherwise specified by the users.

The “Read me first” panel also provides information about the different *D-score* algorithms, the blocks order for the *D-score* computation (i.e., MappingB−MappingA), and the downloadable file that can be retrieved at the end of the computation. Further details on the downloadable file are given in Section 4.5. The blocks order for the *D-score* computation can be changed by reversing the Mapping A and Mapping B labels (see section 4.2).

### 4.2. Input Panel

In the starting state of the app, none of the buttons are enabled, and the input drop-down menus for labeling the blocks are empty. The app comes with a toy data set that can be used to familiarize with the app functions, and that can be uploaded by clicking on the checkbox Race IAT dataset. Users can upload their own data by means of the **Browse** button.

Two different states of the “Input Panel” are illustrated in Figure 1.

Figure 1A depicts the app state when the data set has been correctly uploaded and read by the app. The name of the uploaded file and its extension appear right next to the **Browse** button. The labels of the four different blocks, as they are named in the data frame, are shown into their—alleged—positions (i.e., “MappingA practice block label,” “MappingA test block label,” “MappingB practice block label,” “MappingB test block label” in Figure 1A). In case the uploaded data set has some problems, like it uses another column separator than the comma, the app will not be able to distinguish between the columns, and the drop-down menus for the assignment of the blocks labels will be empty. Users can redefine the labels for each block by clicking and selecting from the drop-down menus. To reverse the direction of the *D-score*, and hence the interpretation of its meaning, users can switch the labels for Mapping A and Mapping B. The **Prepare Data** button becomes active when the data are correctly uploaded and the labels for each level of the IAT blocks are defined. Once the **Prepare Data** button has been clicked and data are ready for the *D-score* computation, the alert message “Waiting for data” becomes “Data are ready,” and the **Select your D** drop-down menu is enabled (Figure 1B). A brief description of the *D-score* algorithms is given next to each option.

The IAT administration procedure of the example data set did not include a built-in correction strategy, and hence a *D-score* algorithm with an *ex post* strategy for the error responses was chosen, specifically the *D3* one. Since the default direction for the *D-score* computation is (MappingB−MappingA), positive scores stand for faster response times in associating Milk chocolate with negative words and Dark chocolate with positive words.

The **Calculate & Update** button and the **Graphic display** options become active after a *D-score* has been selected, as well as the **Accuracy cleaning** option and the **Fast participants cleaning** option. The **Accuracy cleaning** option refers to the elimination of participants with an high percentage of incorrect responses in at least one of the two associative conditions, either Mapping A or Mapping B (Nosek et al., 2002). The default threshold is set at 25%, and participants with an error percentage exceeding this threshold are discarded. Users can modify the default threshold via the **Error percentage** option (active only when the “Yes” option of **Accuracy Cleaning** is selected). The **Fast participants cleaning** refers to the elimination of participants with more than 10% of trials with responses faster than 300 *ms* (Greenwald et al., 2003). If one of these options is selected, the results of the participants meeting these elimination criteria are not displayed in the “D-Score results” panel, but their *D-score*s, and the information on their performance, will still be available in the downloadable file.

The **Download** button is enabled after the first *D-score* is computed.

### 4.3. D-Score Results Panel

**Figure 2:**
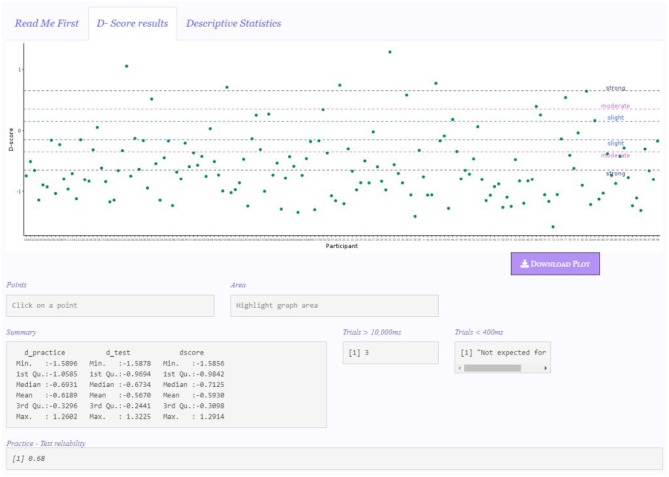
Results panel.

When the **Calculate & Update** button is clicked, results appear in the “D-score results” panel (Figure 2). The **Calculate & Update** button must be clicked every time users want to make a settings change effective, otherwise the app will not be updated.

Despite not shown in Figure 2, the “Input Panel” remains visible on the left side, so that users can constantly check the specific configuration for the computation of the *D-score*.

The first object appearing in this panel is the graphical representation of the results, for which various options are available (“Points,” “Histogram,” “Density,” “Histogram + Density,” see section 4.3.1 for further details). The functioning of the Points and Area boxes is illustrated in section 4.3.1 as well. The default graph appearing is a points graph depicting each participant's *D-score*.

In the Summary box, the descriptive statistics (i.e., *Minimum, First Quartile, Median, Mean, Third quartile*, and *Maximum*) of *D-practice, D-test*, and *D-score* are presented. The Trials >10, 000 ms box reports the number of trials discarded because of a response time higher than 10,000 *ms*. If no trials meet this elimination criterion, the message “None” is displayed. When a *D-score* algorithm that eliminates trials faster than 400 ms (i.e., *D2, D5, D6*) is selected, the Trials < 400 ms box reports the number of discarded trials, otherwise the “Not expected for this D” message is shown, as in the example. Finally, the Practice-Test reliability box shows the IAT reliability computed as the correlation between *D*_practice_ and *D*_test_ across all participants (Gawronski et al., 2017).

Figure 2 depicts the app appearance when the default settings are used (e.g., no participants are discarded, the plot of the *D-score* is the default representation plot). However, users are given the chance to customize the settings configuration for the *D-score* computation, and the display of the results, according to various criteria. For instance, if the **Accuracy cleaning** option is selected, the box Accuracy deletion would appear, reporting the number of participants with an error percentage higher than the selected threshold (if any). Likewise, if the **Fast participants cleaning** option is selected, the box Participants < 300 ms appears, reporting the number of participants with more than 10% of responses with latency faster than 300 ms (if any).

By looking at the graphical representation and the summary statistics of the results, it pops out that respondents' tended to have a preference (dislike) for Milk (Dark) chocolate, since they tended to be faster in Mapping B associative condition (i.e., the condition in which Milk chocolate was associated with positive words and Dark chocolate was associated with negative words). Moreover, the majority of the *D-score*s tended to have a strong effect (see section 4.3.1).

#### 4.3.1. Graphic Display

**Figure 3:**
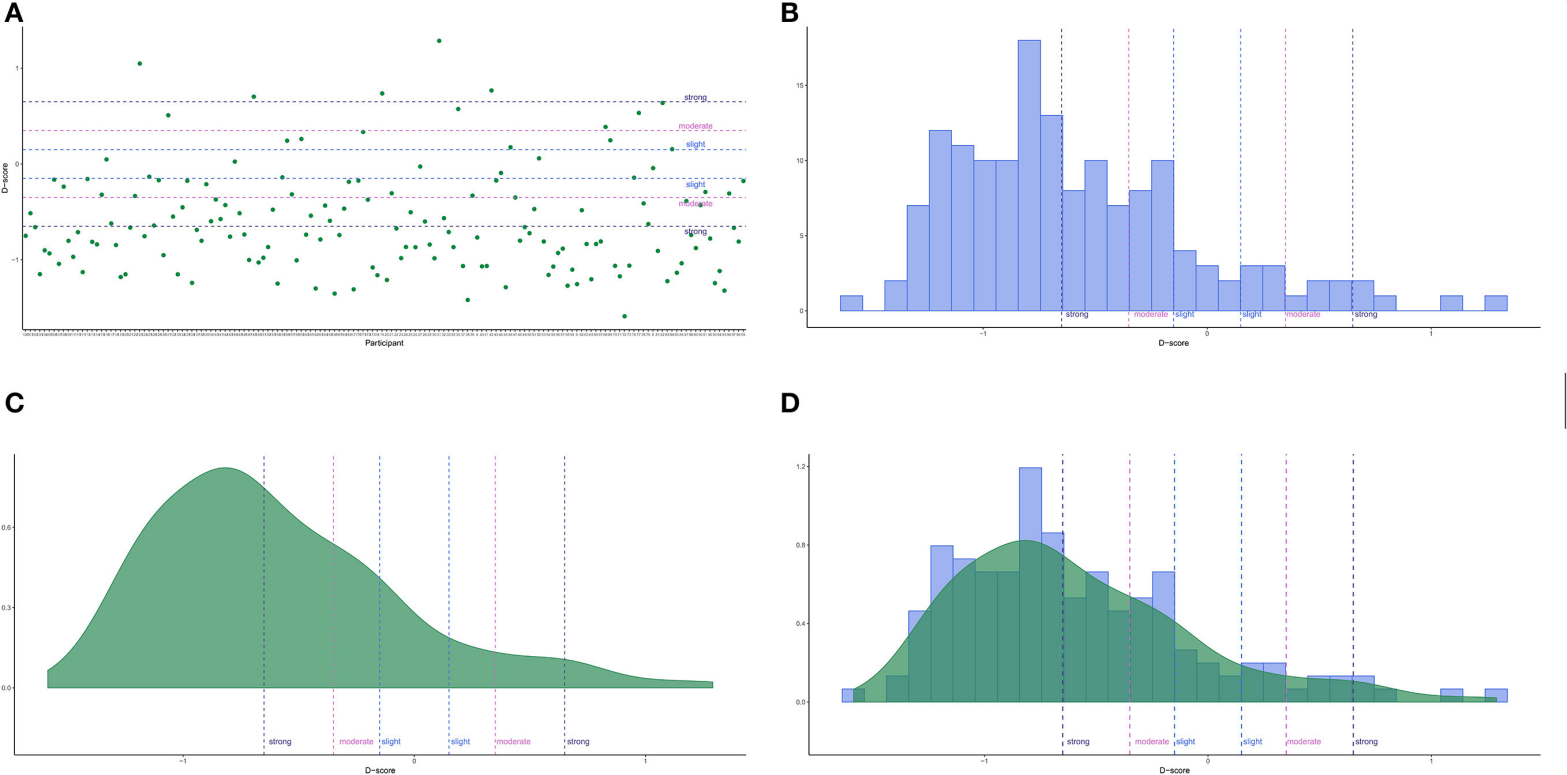
Shiny App graphical representations. **(A)** Points (default). **(B)** Histogram. **(C)** Density. **(D)** Histogram and Density.

DscoreApp provides the possibility to visually inspect the *D-score* results (Figure 3), both at the individual level (Figure 3A) and at the sample level (Figures 3B–D). The lines for interpreting the *D-score*s effect sizes are drawn at ±0.15 (“slight”), at ±0.35 (“moderate”), and at ±0.65 (“strong”), consistently with the guidelines in Project Implicit Website.

Users can customize the graphs to have a better inspection of the results. For instance, in the point graph participants order can be arranged by changing the options in the **Point graph** drop-down menu. The default representation (“None”) follows the order participants had in the original data frame, while the “D-score: Increasing” and “D-score: Decreasing” options arrange participants by increasing or decreasing *D-score*, respectively. In the graphs including the histogram representation (Figures 3B,D), users can set the number of displayed bins by means of a slider, which appears only when either the “Histogram” or the “Histogram + Density” options are selected.

Graphical representation is a convenient way for inspecting the results, particularly for identifying extreme scores. However, it might be difficult to pinpoint a particular score in the graph, and then to link it to the corresponding participant in the data set. DscoreApp provides two useful and handy tools for linking specific points or area of the graphs to the corresponding participants and their *D-score*s. By clicking on a point in the points graph, the ID and the *D-score* of the participant corresponding to that point will appear in the Point box. By selecting an area in any of the graphs, the IDs and *D-score*s of the participants in the selected area will appear in the Area box.

All the graphs are downloadable by clicking on the **Download graph** button, which will be active only after the first graph is displayed. The default name of the graph will contain the type of graph and the specific *D-score* it shows. In the example in Figure 2, the default name will be “PointDefaultDscore3.pdf.” All the graphs have a.pdf extension.

**Figure 4:**
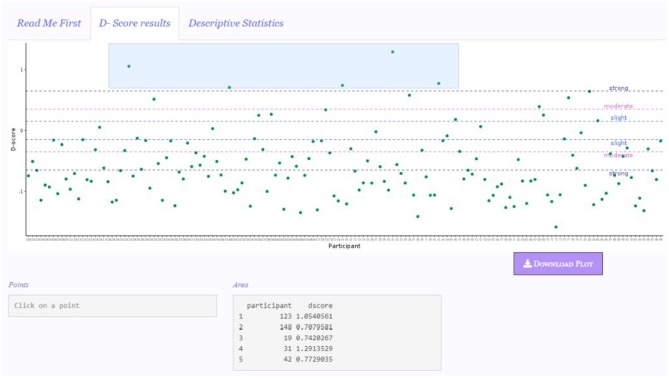
Area highlighter for detecting participants' *D-score*.

In the depicted example, five participants showed a *D-score* far from other participants' *D-score*s. By using the area highlighter, as illustrated in Figure 4, it is possible to immediately and conveniently identify the IDs of these participants (see Area box in the figure), and to check for any particular response pattern resulting in these scores in the original data set. Within these five participants, it is possible to note that there is also the participant obtaining the maximum *D-score* of the sample, namely Participant 31 (see Summary box in Figure 2).

### 4.4. Descriptive Statistics Panel

**Figure 5:**
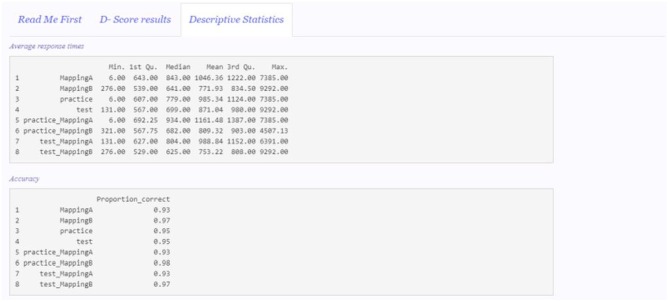
Descriptive statistics panel.

Figure 5 depicts the appearance of the “Descriptive statistics” panel.

The average response times and the proportion of correct responses in each of the mapping conditions and blocks of the IAT are reported. MappingA and MappingB include all the trials in both practiceMappingA and testMappingA and practiceMappingB and testMappingB, respectively. Practice blocks trials (practiceMappingA and practiceMappingB) compose practice, while test blocks trials (testMappingA and testMappingB) compose test. All the other categories (i.e., practiceMappingA, practiceMappingB, testMappingA, and testMappingB) are composed by their respective number of trials in users' original data set.

The descriptive statistics are computed on the same data set on which the *D-score* is computed. For instance, if a *D-score* algorithm with the lower tail treatment is selected, the descriptive statistics are computed without considering the discarded trials. Likewise, if participants cleaning is selected, the descriptive statistics will not include the discarded participants.

### 4.5. Downloadable File

At the end of the computation, users can download a CSV file containing the last computed *D-score*. The default name of the file will contain the number of the selected *D-score* algorithm. The variables contained in the downloadable file are illustrated in Table 4.

**Table 4:** Content of the Downloadable File.

**Variable**	**Content**
participant	Participants' IDs.
n_trial	Number of IAT trials (before data cleaning).
slow10000	Number of trials with latency > 10, 000 ms.
num.300	Number of trials with latency < 300 ms.
num.400	Number of trials with latency < 400 ms.
mean.tot	Average response time across all blocks.
p_correct_block.practice.MappingA	Proportion of correct responses in practice block of Mapping A.
p_correct_block.practice.MappingB	Proportion of correct responses in practice block of Mapping B.
p_correct_block.test.MappingA	Proportion of correct responses in test block of Mapping A.
p_correct_block.test.MappingB	Proportion of correct responses in test block of Mapping B.
p_correct_bpool.practice	Proportion of correct responses in practice blocks (practiceMappingAand practiceMappingB).
p_correct_bpool.test	Proportion of correct responses in test blocks (testMappingAand testMappingB).
prop_correct_cond_MappingA	Proportion of correct responses in Mapping A.
prop_correct_cond_MappingB	Proportion of correct responses in Mapping B.
p_correct_tot	Overall proportion of correct responses.
d_practice.#	*D-score* for the practice blocks.
d_test.#	*D-score* for the test blocks.
dscore.#	*D-score*.
cond_ord	Order of presentation of the two associative conditions (i.e., MappingA_firstor MappingB_first).
LegendMappingA	Users' data set labels for Mapping A (e.g., practiceMappingA_and_testMappingA).
LegendMappingB	Users' data set labels for Mapping B (e.g., practiceMappingB_and_testMappingB).

The value in each column refers to the observed value for each participant. The # represents the number corresponding to the selected *D-score* algorithm.

In the depicted example, the default file name will be “ShinyAPPDscore3.csv.”

## 5. Final Remarks

The user-friendly and intuitive interface of DscoreApp makes its use straightforward, with no need for programming skills. Furthermore, the preparation of the data set for the analyses does not require any particular software or skill.

Beyond making the *D-score* computation easier, DscoreApp provides unique features that are not accessible with the available options for the *D-score* computation. First, DscoreApp provides the ability to immediately see the results and how they change in response to users' configurations. Additionally, since all the important information on participants performance and IAT functioning are available at the same time (e.g., *D-score*s, number of fast trials, IAT reliability), this app allows for grasping a complete overview of the functioning of the IAT. For instance, it allows for an immediate glimpse of how fast trials or inaccurate participants influence the results, and to identify critical aspects of the IAT that might deserve further investigation. Moreover, the preparation of the data set itself is particularly easy: Users will just have to eliminate the pure practice blocks and to rename the columns according to the instructions.

The downloadable file contains all the information that might be needed for further analysis on the IAT, or for plotting the results according to users' needs.

DscoreApp is constantly updated by the Authors, and new functions that are not present in this paper might be available in the future (e.g., other IAT reliability indexes). DscoreApp has been tested on several browsers (i.e., Google Chrome, Safari, Firefox, and Internet Explorer), and it has been found to have a reliable functioning. Problems encountered when using these browsers might be attributable to browsers security settings and/or poor internet connection.

## Data Availability Statement

The code used for the development of Dscore app can be found at OttaviaE/DscoreApp.

## Ethics Statement

Ethical review and approval was not required for the study on human participants in accordance with the local legislation and institutional requirements. The patients/participants provided their written informed consent to participate in this study.

## Author Contributions

OE was responsible for DscoreApp development and for the initial draft of the contribution. PA and ER revised the initial draft of the paper and provided useful comments for the final version.

### Conflict of Interest

The authors declare that the research was conducted in the absence of any commercial or financial relationships that could be construed as a potential conflict of interest. The reviewer AC declared a shared affiliation, with no collaboration, with the authors to the handling editor at the time of the review.
